# The influence of sex on neuroimmune communication, pain, and physiology

**DOI:** 10.1186/s13293-024-00660-w

**Published:** 2024-10-22

**Authors:** Shevon N. Alexander, Audrey R. Green, Emily K. Debner, Lindsey E. Ramos Freitas, Hanna M. K. Abdelhadi, Thomas A. Szabo-Pardi, Michael D. Burton

**Affiliations:** Neuroimmunology and Behavior Laboratory, Department of Neuroscience, School of Behavioral and Brain Sciences, Center for Advanced Pain Studies, University of Texas at Dallas, 800 W. Campbell Road, BSB 10.537, Richardson, TX 75080 USA

**Keywords:** Neuroimmune, Sex differences, Pain, Physiology, Hormones, Translational

## Abstract

With the National Institutes of Health’s mandate to consider sex as a biological variable (SABV), there has been a significant increase of studies utilizing both sexes. Historically, we have known that biological sex and hormones influence immunological processes and now studies focusing on interactions between the immune, endocrine, and nervous systems are revealing sex differences that influence pain behavior and various molecular and biochemical processes. Neuroendocrine-immune interactions represent a key integrative discipline that will reveal critical processes in each field as it pertains to novel mechanisms in sex differences and necessary therapeutics. Here we appraise preclinical and clinical literature to discuss these interactions and key pathways that drive cell- and sex-specific differences in immunity, pain, and physiology.

## Abstract (plain language summary)

In the last decade, NIH-funded basic and clinical research studies have been mandated to include sex as a biological variable, and this has resulted in a boom of studies discovering sex differences. We know that females are more likely than males to develop chronic pain conditions and experience higher levels of pain for longer periods of time. Conditions that demonstrate this include migraine, arthritis, and peripheral neuropathy. Furthermore, these sex differences extend to surgical outcomes and are observable at the molecular, cellular, and systemic levels. Although pain perception pathways differ by sex, studies are also focusing on differences in the “conversation” between the immune system and the nervous system while addressing implications of sex hormones to gain a better understanding of the impact on pain, physiology, and behavior. Lifestyle factors such as diet and alcohol consumption also play a role in affecting the perception and impact of pain conditions. As this area of research continues to grow, the development and availability of sex-specific treatment options will grow and lead to improved patient outcomes and more effective pain management strategies.

## Highlights

- Immune cell types and molecular pathways drive sex specific differences in inflammatory and neuropathic pain.
- TLR4 involvement in pain pathways show contextual sex-specific variations and its interaction with TRPV1 channels adds complexity to nociceptor physiology.
- Female mice tend to exhibit stronger TRPV1-mediated pain responses compared to males due to estrogen enhancement of TRPV1 expression levels.
- The interplay between nuclear receptors (PPARγ, PPARα) and prolactin receptors influences sex-specific neuroimmune interactions. In males, PPARα impacts immune responses and neuroinflammation. In females, PPARγ activity influences pain perception and PRL influences pain sensitivity.
- Personalized approaches are needed for pain management in males and females due to sex differences in pain perception and treatment outcomes driven by variations in hormone levels, adiposity, and neuroimmune mechanisms of inflammation.

## Neuroimmune interactions, pain, and sex differences: an overview

The study of immune-to-neural interactions, or neuroimmunology, is a burgeoning field that integrates two of the most complex organ systems of the body. With roots in immunology and neuroscience, this integration spans over 45 years of studies with influence from physiology investigators like Ivan Pavlov that hypothesized that immunity could be conditioned, and Hans Selye, who demonstrated that various stressors influence immune and endocrine effects [1–5]. Researchers in the 1970’s coined the term “psychoneuroimmunology” and put this research topic on the map as a pertinent integrative research necessity [6–8]. Over the years, the field has split into defined categories with psychology and molecular neuroscience dictating the major research focus arms [9–11]. A surge in interest and citations in immune-to-neural interactions happened in 2014 and coincided with the national institutes of health’s (NIH) mandate that all funded studies needed to consider sex as a biological variable (SABV) [12]. This is a logical extension because the integrative nature of neuroimmunology allows for the assessment of both behavioral and physiological output that can validate the influence of one system over the other and any apparent sex differences. This is a unique aspect of the field where the inherent experimental design allows for our understanding of these interactions and outputs at various levels (Table 1).

**Table 1:** Summary of the described sex differences across various immune-, neuro-, and endocrine-based targets. The denotation of “N/A” highlights interactions and mechanisms that have yet to be elucidated and posits the exploration of whether sex differences exist for these pathways

Target	Sex Differences		References
	Males	Females	
TLR4	• Global deletion of TLR4 attenuates post-inflammatory allodynia. • Intrathecal injection of a TLR4 antagonist reverses pain-like behavior in arthritis. • TLR4 on macrophages drives inflammatory pain.	• TLR4 on neurons drives neuropathic pain.	Woller et al., [208]; Christianson et al., [209]; Szabo-Pardi et al., [151]
Macrophages	• Pro-inflammatory characterized by M1 states. • Male-biased roles in inflammation and pain.	• Anti-inflammatory and modulated by estradiol.	Lenert et al., [170]; Yu et al., [182], Rudjito, R., et al., [246]
Microglia	• Pro-inflammatory phenotype characterized by amoeboid morphology and an inflammatory transcript profile. • Male-specific role in mediating the development of chronic neuropathic pain.	• CSF1 mediates crosstalk between lymphocytes and spinal microglia thus playing a role in sex-specific differences in neuropathic pain. Microglia TLR4 drives female-specific allodynia after short-term alcohol.	Lei et al., [295]; Sorge, [183]; Sorge, [166]; Inyang, [167]; Agalave [207]; Kuhn et al., [174]; Alexander et al., [233]
T-cells	• Pain responses characterized by neutrophil recruitment, monocyte infiltration into the CNS, and activation of microglia for neuro-immune crosstalk.	• Greater involvement in female pain development and outcomes. • In neuropathic injury models, T-cell infiltration into the spinal cord resolved pain in females but not males. • Females lacking T-cells do not experience pregnancy analgesia in neuropathic or inflammatory pain models.	Sorge et al., [166]; Mifflin et al., [172]; Chernov et al., [173]; Scheff et al., [165]; Kuhn et al. [174]; Rosen et al., [261]
Estradiol	• N/A	• Act on macrophages, which consequently increases the expression of toll-like receptors. • Play a critical role in anti-inflammatory processes, but they can also have pro-inflammatory effects • Low estradiol levels correspond to increased pain perception. ER-α inhibits pain transmission while ER-β promotes pain transmission	Lenert et al., [248]; Smith et al., [282]; Paredes [286], Tang et al. [278]; Zhong et al. [277]; Li et al. [330]
Testosterone	• Decreases levels of pro-inflammatory cytokines, IL-1β and TNF-α, and an increase in the anti-inflammatory cytokine, IL-10 • Increased concentrations lead to decreased pain outcomes	• Activated androgen receptors regulate the expression of TRPV1 channels which decreases pain	Malkin et al., [269]; Edinger and Frye [262] & [263]; Lee [264]; Zhou [265]
Progesterone	• N/A	• Decrease inflammation by inhibiting pro-inflammatory cytokine production and increasing anti-inflammatory cytokines • Mediates neuropathic pain. Peaks in progesterone are associated with lower pain severity associated with fibromyalgia	Klein and Flanagan, [292]; Schertzinger et al., [21]
Prolactin	• N/A	• Increased levels play a role in autoimmune diseases • Prlr in sensory neurons are critical for developing pain from the acute to chronic states in females only	Shelly et al., [309]; Paige, et al., [312]
Fc Receptors	• N/A	• Mediates pain independently of immune cell involvement and inflammation	Qu et al., [159]; Bersellini Farinotti et al., [160]; Goebel et al., [158]; Liu et al., [155]; Jiang [156]; Wang [157]
TRPV1	• Testosterone inhibits TRPV1 expression in inflammatory pain	• Positively activated by estradiol which suppresses production of cytokines by tissue resident macrophages • Pain outcomes influenced by estradiol concentrations	Bai et al., [265]; Chen et al., [252]; Wu et al., [284]; Cho and Chaban, [251]; Yamagata et al., [285]; Payrits et al., [249]
PPARα	• Protective and anti-inflammatory • Activation promotes pain relief	• N/A	Zhang et al., [316]; Sorge et al., [260]
PPARγ	• N/A	• Estradiol promotes anti-inflammatory processes in macrophages • Activation promotes pain relief	Zhang et al., [316]; Sorge et al., [260]

**Fig. 1:**
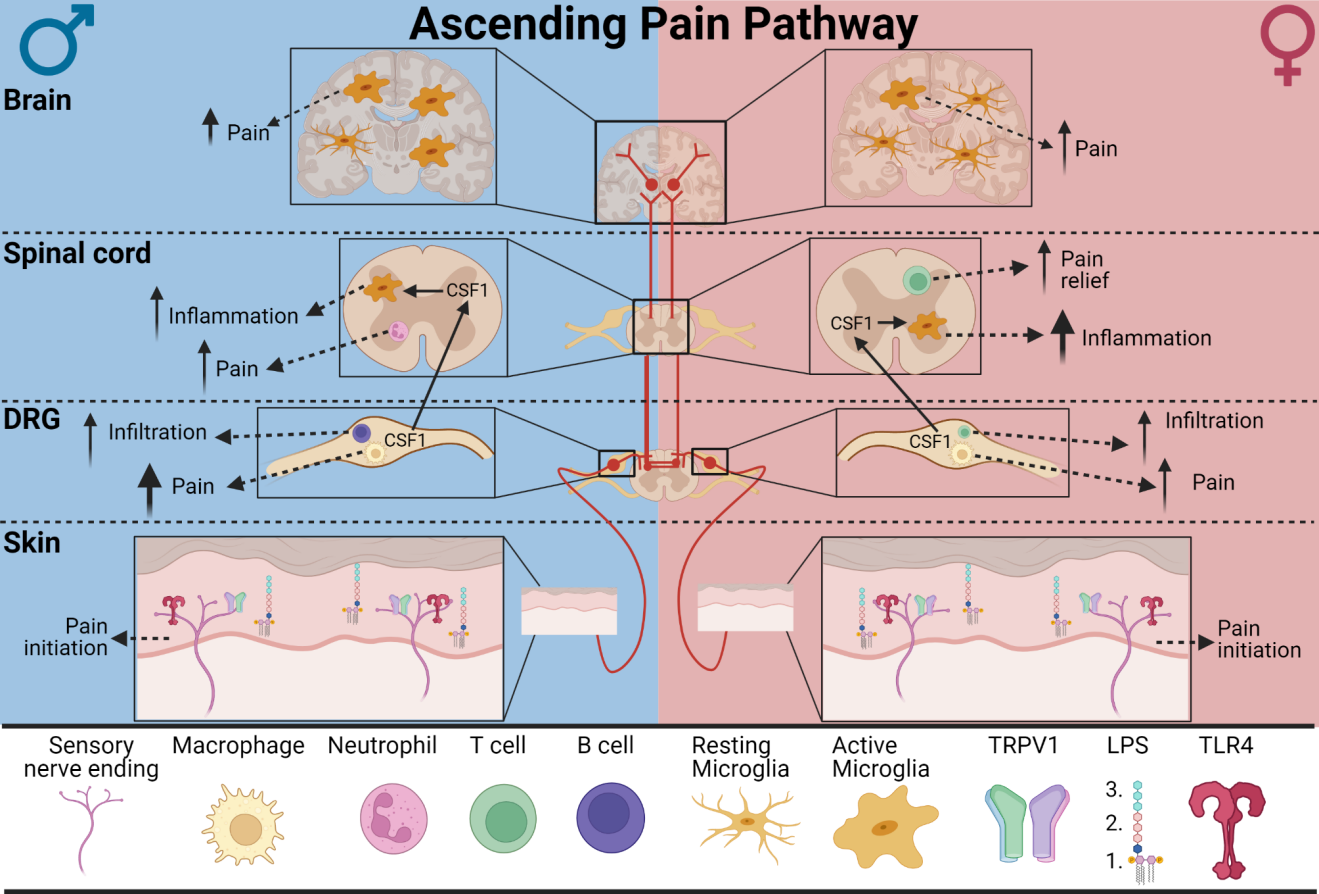
The ascending pain pathway (Red). Noxious stimuli, such as thermal, chemical, or mechanical stimuli, or inflammatory stimuli, such as lipopolysaccharide (LPS), are detected by receptors expressed on nociceptors innervating peripheral tissue. Thermal stimuli activate TRP channels, such as TRPV1, while inflammatory stimuli activate other receptors, like activation of TLR4 by LPS. Following stimulation, nociceptors initiate pain transmission by propagating information to sensory neuron cell bodies located in dorsal root ganglia (DRG). These neurons synapse onto second order neurons within the dorsal horn of the spinal cord which in turn transmit nociceptive signals to the brain where it is interpreted as pain. Neuroimmune interactions within different levels of the ascending pain pathway influence the development and maintenance of pain in a sex- and cell-specific manner. Differences in arrow sizes indicate level of contribution, with larger arrows demonstrating greater impact, and are used to highlight sex-specific differences

One area that has gained interest is how the nervous system and immune system communicate to modulate pain and how pain differs between sexes. Pain is a multi-faceted, subjective experience that involves the integration of peripheral sensory systems and higher brain processing centers. Within the peripheral nervous system (PNS), the dorsal root ganglia (DRG), which are a heterogeneous population of sensory neurons, innervate peripheral tissues and are the first level of integration of sensory information and mediation of responses to noxious stimuli. These nociceptive neurons, or nociceptors, are characterized by their varying degrees of myelination, size, and signaling capabilities [13]. Recent single-cell RNA-sequencing data indicates that there are at least 10 major populations of sensory neurons in the murine DRG, with numerous subtypes that further distinguish their unique physiological properties [14]. Further, spatial transcriptomics have identified at least twelve sensory neuron subtypes in human DRG [15, 16]. These neurons actively respond to different external stimuli, such as changes in thermal, mechanical, or chemical stimuli. They play a necessary role to alert the individual to potentially harmful situations or environments and initiate defensive behaviors. When activated by a noxious stimulus, DRG nociceptors transduce this signal to the dorsal horn of the spinal cord and then to the brain, including the periaqueductal gray, amygdala, hypothalamus, and other regions of the cerebral cortex, to be processed and interpreted as pain (Fig. 1).

Short term, or acute pain, can prompt defensive behaviors following injury to facilitate healing. Maladaptive plasticity of the nervous system can translate to various pain states [17]. Long-term, or chronic pain, is characterized by pain that persists beyond a biological benefit and is a pervasive disease state that affects over 20% of the world’s population, with an additional 10% diagnosed with chronic pain each year [18]. In the United States, the Centers for Disease Control and Prevention (CDC) lists chronic pain as the number one cause of long-term disability and loss of productivity [19]. Chronic pain has a higher prevalence in females, which account for 70% of cases; females experience increased severity and duration of pain compared to males [20]. Females are more likely to develop pain disorders that affect the musculoskeletal system such as fibromyalgia [21], osteoarthritis, low back pain [22], and migraines [23, 24]. Moreover, and to a lesser extent, females have reported more pain after invasive procedures than males [25]. Since pain is such a complex physical and emotional state, preclinical or translational studies assess the physical influence of noxious stimuli, or nociception [26].

One mechanism by which chronic pain occurs in the periphery is ectopic activity of peripheral nerves and cell bodies. During ectopic activity, pain is generated as a result of ion channel and receptor upregulation and hypersensitization to neuromodulators [27]. Specific channel subtypes, such as Transient Receptor Potential Channels (TRP) channels TRPA1 and TRPV1, are crucial components of nociceptor signaling as they regulate pain, temperature, and mechanical sensation through Ca^2+^ influx [28]. Activation of these TRP channels on nociceptors have been implicated in the pathogenesis of chronic pain (Fig. 1). Following injury or inflammation, TRP channels are modulated in 2 ways: (1) their expression in DRG neurons are upregulated and (2) the channels are sensitized through phosphorylation, both of which contribute to enhance neuronal activity and chronic pain [29–31]. This may suggest that TRP channels could be a promising candidate for pain therapeutics [32]. Dysregulation of central nociceptive circuitry can also lead to maladaptive plasticity and chronic pain. Following exposure to certain stimuli, neurons in the central nervous system (CNS) can exhibit increased responsiveness to previously subthreshold inputs from peripheral sensory neurons to enhance pain responses. While intense noxious stimuli elicit pain responses of sensory neurons, it is noteworthy that repeated or prolonged non-noxious stimuli, such as moderate alcohol consumption, short-term high-fat diet, or other dietary components such as ω-6 polyunsaturated fatty acids, also reduce pain thresholds and can contribute to the development of chronic pain [33–35].

The immune system plays a key role in the activation and sensitization of nociceptors. Under inflammatory conditions, such as tissue injury or infection, immune cells release signaling molecules that can stimulate nociceptors to initiate nociceptive signaling or increase expression of receptors for noxious stimuli [36]. Conversely, nociceptive neurons can also regulate immune responses and inflammation [37–39]. The basic mechanisms and dynamics of neuroimmune modulation of pain are under investigation; however, not all is equal between sexes considering cell activity. This review discusses neuroimmune interactions in pain physiology and how these interactions differ between sexes.

## Neuroimmune interactions in pain physiology

Considerable evidence shows that immune cells play a significant active role in the onset and persistence of chronic pain conditions. Tissue resident immune cells inhabit dedicated tissue types and adapt to the metabolic function and defense of the tissue [40–45]. Tissue resident immune cells are the main actors in mediating inflammatory responses that precipitate acute and chronic pain in their efforts to preserve tissue integrity and have been demonstrated to be primary contributors to muscle pain [46]. They directly interact with and receive signals from neurons for rapid and stronger immune responses to danger signals, infections, and physiological imbalance within the tissue.

Immune cells are activated by a variety of substances such as damage-associated molecular patterns (DAMPs) released by damaged cells, cytokines, and chemical messengers released from neurons, including norepinephrine and acetylcholine (Ach), substance P and calcitonin gene related peptide (CGRP) [47–51]. The pro-inflammatory cytokine, Tumor Necrosis Factor-α (TNF-α) can activate and induce glutamate release from microglia in cases of oxidative stress to produce neurotoxic effects on neurons [52–54]. Following their activation, immune cells migrate toward damage signals from within the tissue and provide tissue protection by phagocytosis of pathogens and dead cells and by releasing factors to stimulate repair and regeneration of tissue-specific cells such as epithelial cells, fibroblasts, and neurons [55, 56]. Immune cells, such as DRG resident macrophages and natural killer (NK) cells [57–60]can also directly interact with sensory neurons to mediate pain and sensation. For example, injury to the DRG triggers a pro-inflammatory activation state of macrophages [61, 62]. Consequently, this immune response increases phagocytic activity, activates NK cells, induces cytotoxic action, and propagates pro-inflammatory cytokine release culminating in enhanced neuron responsiveness [57, 59]. This enhanced activity leads to maladaptive plasticity and sensitization of neurons to lower pain thresholds and sustain chronic pain states.

In addition to tissue-resident immune cells, circulating immune cells which travel in the blood and lymph, respond to danger signals throughout the entire body, modulate neuronal activity as part of the humoral response and vice versa through neuroimmune crosstalk [37, 63]. For example, neurons can engage circulating monocytes via expression of the chemokine C-C Motif Chemokine Ligand 2 (CCL2) which binds to C-C Motif Chemokine Receptor 2 (CCR2) on monocytes, recruiting them to sites of injury [36, 64–66]. Tissue infiltrated monocytes differentiate into macrophages and modulate neuronal activity directly. Circulating neutrophils and helper T-cells are also recruited to injury sites and contribute to sensory neuron signaling and sensitization by releasing pro-inflammatory cytokines, such as TNF-α, Interleukin-6 (IL-6), and Interleukin-1β (IL-1β), and other factors [67, 68]. These molecules increase neuronal excitation and alter pain sensitivity. For example, IL-1β binds to DRG sensory neurons and promotes nociceptive signaling by relieving the resting slow inactivation of sodium channels to increase sodium currents and nociceptor excitability [69, 70]. Immune cells also release anti-inflammatory cytokines such as Transforming growth factor-β1 (TGF-β1) and Interleukin-10 (IL-10), which promote sensory neuron repair and reduce neuronal signaling to abrogate pain transmission and contribute to pain resolution [71–73]. While these circulating immune cells can be studied via whole blood isolation and plasma analysis, tissue resident immune cells are much more difficult to study due to tissue accessibility issues and overlapping identifying markers to infiltrating circulating cells [74–77].

Other circulating immune components, such as complement proteins C3a and C5a, contribute to direct neuronal stimulation that leads to pain transduction [78–80]. These two complement proteins, generally referred to as anaphylatoxins, are primarily effectors of innate immunity concerned with the defense of neurons by promoting inflammation [81, 82]. C3a and C5a have a neuroprotective role against excitotoxic neuronal injury, promoting neurogenesis, inhibiting caspase-3 activity, regulation of glutamate receptor expression, and astrocyte stimulation [83–87]. Despite these neuroprotective effects, C3a and C5a can trigger pain responses to heat by direct modulation of neurons via activation of neuronal complement-specific receptors and TRPV1 channels [78, 88].

Important to note, this is a two-way conversation, and not only can immune cells influence neuronal activity, but neurons can regulate the activity of immune cells. Sensory neurons transmit electrochemical signals to lymph tissues and immune cells to modulate inflammatory and pain responses through molecular- and receptor-mediated communication [36, 89–91]. Moreover, new research has demonstrated sensory neurons participate in neuroimmune crosstalk with various tissues that were historically considered passive, including the skin, colon, lungs, and adipose tissue to drive physiology and further drive immunological responses [89, 92–95]. Immune cells express receptors for neurotransmitters that modulate their response to noxious signals and activation of these receptors on immune cells regulates the production and release of inflammatory molecules that can in turn stimulate neurons [96, 97]. For example, GABA suppresses T-cell and macrophage cytokine production capabilities, thus dampening inflammatory responses, and GABA bound to receptors on T-cells produces a profound effect by blocking intracellular calcium signaling and MAPK activation [97, 98].

Both neuronal and immune cell types undergo metabolic changes where neuronal synaptic plasticity is modified, and resident immune cells are activated and transformed morphologically [99, 100]. Further, these signals can mediate a variety of genomic and non-genomic effects that modulate pain and homeostasis [101–105]. Genomic effects are coordinated through intracellular receptors that function as transcription factors and influence mRNA levels for specific proteins such as cytokines, prostaglandins, and other inflammatory mediators. Membrane-bound receptors coordinate both genomic and non-genomic effects, inducing rapid signaling via ion channels, G-protein coupled receptors, and growth factor receptors that activate kinase signaling cascades [101, 106]. Both sensory neurons and immune cells express receptors for a variety of signaling molecules that can initiate these cascades, such as purinergic receptors (P2X/P2Y) [107–110], DAMPs [111], and Toll-like receptors (TLR) [111–113].

One example of neuroimmune communication is neuron-microglia crosstalk in the CNS. Microglia, CNS resident immune cells, survey their environments and monitor neuronal conditions. Activated microglia modulate neuronal activity to drive behaviors, like learning and memory [114–118], depression [119–123], sickness [115, 124, 125], and pain [126–130]. Injury can induce the release of various signaling factors from damaged cells [131–133]. These stimuli activate microglia and attract them to damaged areas to clear debris and dead cells. For example, glutamate levels rise under inflammatory conditions, such as a traumatic brain injury, which increases neuronal activity and contributes to the development of central sensitization and persistent pain. In addition to increasing neuronal activity, glutamate induces calcium influx which initiates ATP-ADP release from neurons leading to stimulation of microglial P2Y_12_ receptors [134]. Microglial P2Y_12_ stimulation is one of the first steps of microglial activation and induces microglia to migrate toward the site of injury. Further, ATP signaling on microglia via P2X receptors increases intracellular Ca^2+^ and triggers the release of cytokines and chemokines and is important for phagocytosis responses [135–137]. Microglia also become activated by neurotransmitters, glutamate, and acetylcholine, via NMDA/AMPA and cholinergic receptors, respectively [135, 138–141]. Glutamatergic stimulation of microglia triggers their modulation of neurons by the release of signaling factors to facilitate neuroimmune crosstalk and by directly wrapping around damaged axons [116, 139, 142–145].

The action of microglia in response to neurotransmitters can be different based on their location in the brain. For example, it has been shown that following treatment with the glutamate receptor-agonist, N-methyl-D-aspartic acid, microglia are neuroprotective in the CA3 and DG regions of the hippocampus, maintaining a ramified state in the CA3 region and “hypertrophic” morphology in the DG [146]. In contrast, in the CA1 region, microglia are not neuroprotective, adopt an amoeboid state, and NMDA- excitotoxicity induces neuronal death [146]. It is possible that these different microglial responses to NMDA may be due to different receptor subtype expression in different regions of the brain and differences in age [147].

## Immune components expressed by neurons to mediate pain

**Fig. 2:**
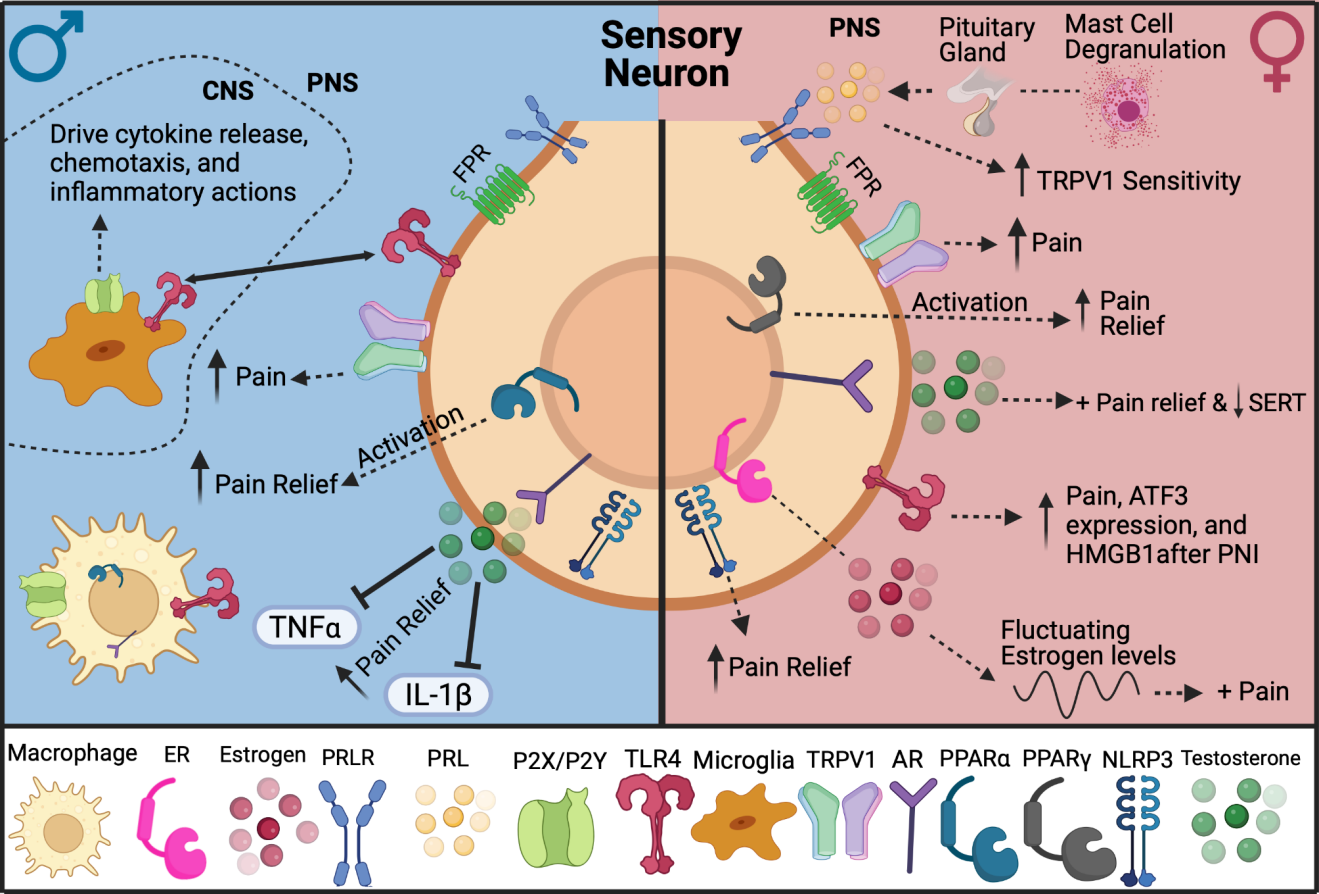
Sexual dimorphic mechanisms in neuroimmune signaling driven by interactions with sensory neurons. In females, sensory neurons are thought to be the main drivers in pain signaling as seen by the female-skewed pathways

Emerging research shows specific receptors and molecules participate in driving pain states differently depending on sex [101, 148]. Additionally, peripheral sensory neurons release neuropeptides, like Substance P and CGRP, that can directly activate nociceptors and enhance infiltration of circulating immune cells to act on injured nerves, causing sensitization and skewing them toward pain states [148–150]. Activation of Toll-Like Receptor 4 (TLR4) and NOD-Like receptor protein 3 (NLRP3) on neurons in response to pathogen-associated molecular patterns (PAMPs) and DAMPs leads to the expression of molecules that promote maladaptive plasticity of neurons and enhance their sensitization toward pain states. In injury models, sensory neuron signaling via TLR4 and NLRP3 lead to greater pain sensitivity in females compared to males, suggesting activation of these receptors produces different cellular responses [151, 152]. Pathogens release molecules, such as N-formyl peptides, that also circulate and directly activate neurons via N-formyl peptide receptors (FPR) to protect against further damage to infected areas [153] (Fig. 2).

Immunoglobulin-like receptors (Fc) are expressed on sensory neurons and are a key component in mediating pain independent of immune cell activation or inflammation [154–157]. Fc receptors interface with circulating immunoglobulin subtypes and facilitate increases in intracellular calcium to sensitize sensory neurons or heighten nociception in females [155, 158–160]. In a study investigating the role of Fc receptor subtypes, FcγRII and FcγRIII, using the K/BxN model of serum-transfer arthritis, only FcγRIII-deficient mice had decreased paw inflammation [161]. Intriguingly, not only was there a lack of attenuation of paw inflammation in the FcγRII-deficient mice, but the time to onset was decreased and the severity of arthritis was increased [161]. Understanding the interactions of these immune components and their direct action on neurons to contribute to pain may highlight them as potential targets for prevention of pain.

## Sex differences in neuroimmune interactions

Through pre-clinical and clinical studies, neuroimmune interactions have been shown to propagate and maintain pain states differentially in males and females [162, 163] An example of this is the NLRP3 inflammasome in inflammatory pain models, which contributes to female-specific, sensory neuron driven pain [152, 164]. In an oral cancer pain model, male-specific sexual dimorphisms were observed in neutrophil-mediated suppression of pain [165]. Microglia in the CNS are reported to possess a more male-specific role in mediating mechanisms in the development of chronic pain [166, 167], but there is new data that suggests that cell signaling has more influence than specific cell types [168].

In females, T-cells have been shown to have a greater involvement in pain development and outcomes [166, 169–173]. In a neuropathic injury model, T-cell infiltration into the spinal cord was shown to resolve pain in females but not males [166, 174]. Furthermore, female mice lacking T-cells did not experience pregnancy analgesia in neuropathic or inflammatory pain models [175].

**Fig. 3:**
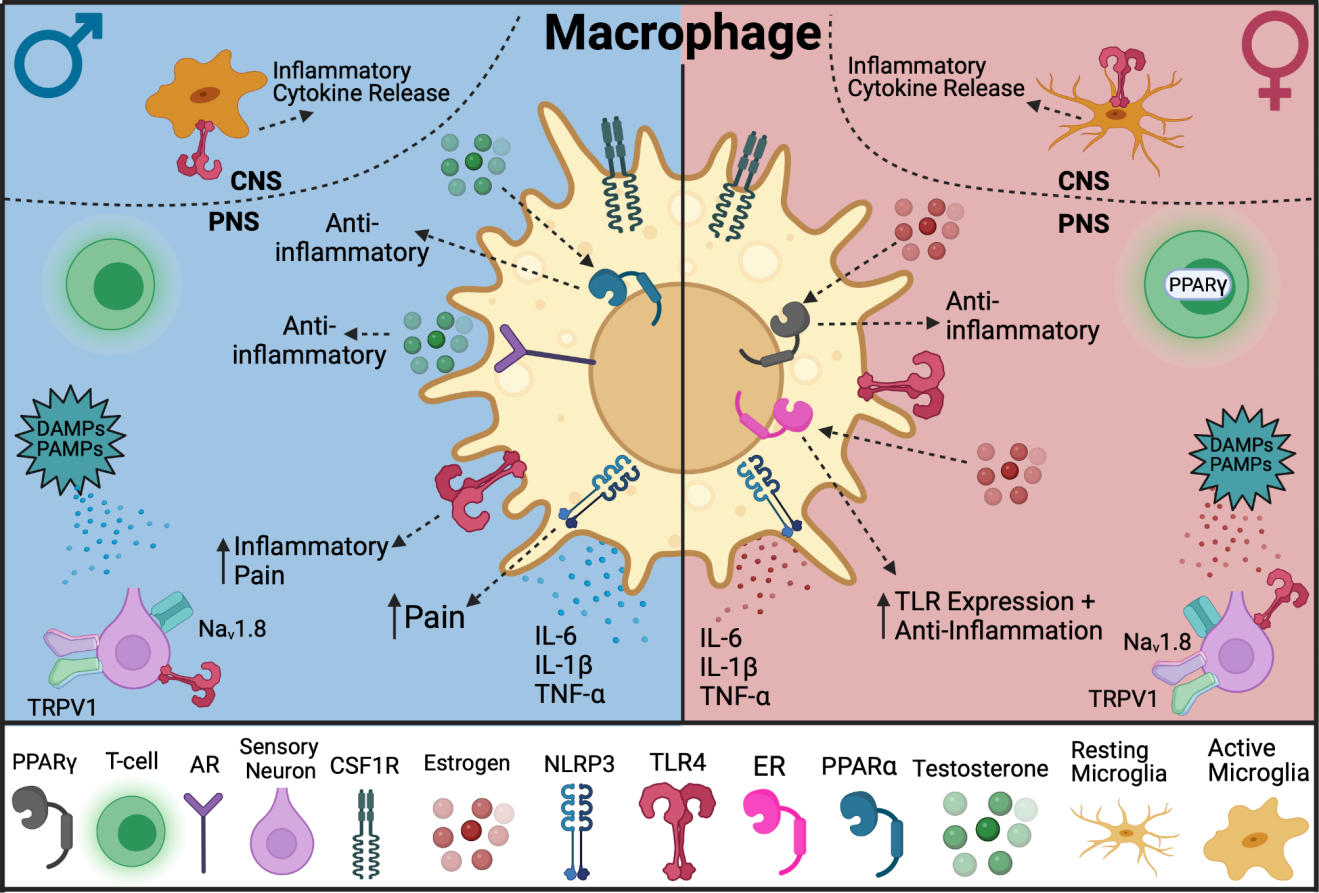
Sexual dimorphic mechanisms in neuroimmune signaling driven by interactions with macrophages. In males, immune cells are thought to be the main drivers in pain signaling, such as through TLR4 signaling

A study that compared central and peripheral immune cell infiltration following nerve injury suggests sex dimorphic processes in the adaptive immune cells that infiltrate the DRG 8 days following injury, with greater B-cell infiltration in male mice and greater T-cell infiltration in female mice [176]. Peripheral nerve injury increases inflammation and pain not only at the site of injury but also in the central nervous system. One mechanism for this spread of pain signals involves colony-stimulating factor 1 (CSF1), a cytokine important for macrophage differentiation. Following peripheral neuropathic injury, CSF1 is expressed *de novo* in the DRG. CSF1 then travels to the spinal cord where it interacts with microglia in the dorsal horn [177]. Interestingly, research suggests that CSF1 is expressed at higher levels in females than males after neuropathic injury suggesting a sex-dependent regulation of CSF1 signaling during pain development [178]. While studies show male-specific responses to intrathecal CSF1 administration, some lack data on female responses, making direct sex comparison difficult [177, 179]. This raises the possibility that CSF1 signaling on a cellular level may be similar in both sexes, but in females, higher levels of CSF1 may contribute to differences in neuropathic pain. Additionally, if CSF1 expression levels is indeed the key to sex disparity, using the same intrathecal dose of CSF1 may mask sex differences in neuropathic pain models. Intrathecal CSF1 has shown a male-biased outcome due to microglia’s role in regulating pain states in males [127, 180, 181]. CSF1 can mediate crosstalk between lymphocytes and spinal cord microglia in females by acting as a modulator of Treg immunosuppressive activity [174]. Peripheral macrophages also express the receptor for CSF1 (CSF1R) (Fig. 3), suggesting that macrophages in addition to microglia play a role in sex-specific differences in CSF1-mediated neuropathic injury. More evidence for neuroimmune crosstalk, was described when deletion of the CSF1 ligand in sensory neurons reduced tissue resident macrophage proliferation (Ki67^+^) in the DRG of males but not females following neuropathic injury [182].

Additionally, other receptors such as TLR4 on different immune and neuronal populations can lead to differential pain sensitivity in males and females [183]. A study has shown that TLR4 on nociceptors drives early neuropathic pain development in a female-specific manner [151]. These data along with the NLRP3 data imply sensory neuron-derived mechanisms differentiate male and female pain development. The involvement of these receptors and immune cells to facilitate neuroimmune communication can help to determine potential mechanisms underlying pain and immunity.

## TLR4: a mediator of sex-specific differences in neuroimmune interactions

Toll receptors are evolutionarily conserved proteins involved in innate immune processes that recognize patterns ranging from lipopolysaccharide (LPS) to nucleic acids such as viral RNA and CpG DNA [184–188]. The subtype TLR4 forms a multi-receptor complex on the surface of a cell with several other proteins and adaptors. LPS, TLR4’s primary agonist, is comprised of 3 primary structure domains: (1) the lipid A tail, (2) the core oligosaccharide, and (3) the O antigen (Fig. 1) [189]. The hydrophobic lipid A tail is a motif that is recapitulated in not only pathogens, but DAMPs produced in vivo, and the backbone of fatty acids consumed in the western diet [34, 190, 191]. TLR4 is expressed on cells in both the CNS and periphery including microglia [192], astrocytes [193], neurons [194], macrophages [195, 196], endothelial cells [197], fibroblasts [198] and more. Since TLR4 is expressed on a variety of cells with specific biological functions, considerations must be made on a per cell basis when interpreting the effects of TLR4 signaling in disease pathology.

Evidence suggests that in the DRG and trigeminal ganglia (TG), TLR4 and TRPV1 are functionally coupled and TLR4 can directly modulate activity of TRPV1 [199, 200]. Infusion of LPS on dissociated TG and DRG neurons potentiated TRPV1 agonist responses such as calcium influx and CGRP release, both of which are not directly linked to TLR4 signaling cascades [201]. In TLR4 knockout mice, TRPV1 agonist-induced pain is significantly reduced [199]. Moreover, TLR4 possesses the ability to modulate sodium channel (Na_v_) activity—an essential component of nociceptive transmission. Upon activation, TLR4 initiates an intracellular signaling cascade that activates NF-κB which interacts with pain sensitive Na_v_s on nociceptors within minutes of activation to increase Na_v_ currents and decrease recovery time from inactivation, which enhance neuronal excitability and pain signaling [202]. Taken together, these studies reveal a unique interaction between TLR4 and TRPV1 as well as TLR4 and Na_v_s, which enhance the context by which we understand nociceptor physiology.

## Specific modulation of pain by TLR4

Evidence has mounted that TLR4 is a critical mediator of the nervous and immune system’s inflammatory response during pain [91, 202–204]. TLR4 gene expression is increased in the spinal cord when rats are injected with intraplantar CFA [205]. Subsequent studies have verified this by showing an increase in microglia reactivity in the dorsal spinal cord following intraplantar CFA injection [206]. Another study also demonstrated the involvement of TLR4 in acute inflammatory pain by utilizing intrathecal injections of high mobility group box 1 (HMGB1), a TLR4 agonist, which enhanced activation of microglia in a TLR4-dependent manner subsequently producing hypersensitivity in mice [207].

TLR4 participates in several inflammatory conditions that have previously been found to differ by sex. For example, rheumatoid arthritis, has a more pronounced effect in women [208]. In a rodent model of arthritis, it was revealed that while both females and males demonstrate allodynia after inflammation, males continue to have tactile allodynia after joint inflammation has resolved [208]. However, global deletion of TLR4 in both sexes attenuated the post-inflammatory allodynia [208]. In a similar model, a global deletion of TLR4 attenuated the post-inflammatory allodynia in males, whereas an intrathecal injection of a TLR4 antagonist given during the inflammatory phase fully reversed the pain-like behavior associated with arthritis in males [209]. These findings suggest that TLR4 signaling alone can sustain the inflammation seen in arthritis and contributes to sex differences in the manifestation of the disorder.

In addition to inflammatory pain, TLR4 has also been shown to be involved in chemotherapy-induced peripheral neuropathy (CIPN). CIPN is one of the major dose-limiting side effects of cancer treatments. Paclitaxel, a taxane-based chemotherapeutic, engages similar inflammatory intracellular signaling pathways as the classical TLR4 ligand, LPS [210]. This is evident by paclitaxel utilizing the same accessory protein required for LPS signal transduction and upregulating inflammatory cytokines such as IL-1β [211]. Paclitaxel treatment upregulates TLR4 expression in the DRG and spinal cord, suggesting its connection with the development of pain [212], and blocking TLR4 signaling reverses this pain phenotype [213].

TLR4 plays a significant role in opioid-induced hyperalgesia [214, 215]. While this seems paradoxical, evidence suggests that µ*-*opioids can lead to increased pain sensitivity [216]. It is believed that certain opioids can bind to TLR4, which facilitates inflammatory signaling [217]. By acting as TLR4 ligands, traditional opioid receptor agonists induce a pain phenotype or enhance sensitivity [218].

## Sex differences in TLR4-mediated pain development and allodynia

Over the last decade, TLR4 has made its way into the spotlight of pain research. However, its exact sex- and cell-specific role in mediating chronic pain development and maintenance have been controversial. One of the first studies to implicate TLR4 signaling in neuropathic pain development sought to characterize the functional role of microglia TLR4 in response to neuropathic injury [219]. They found that TLR4 whole body null mice had an attenuated spinal microglial inflammatory response, but also a reduced nociceptive behavioral phenotype after nerve injury. Subsequent studies have corroborated these findings by pharmacologically inhibiting spinal TLR4 activation [220, 221]. While these studies do provide convincing evidence that suggests immune-mediated TLR4 signaling contributes to neuropathic pain development, sex was not considered as a variable. It was not until a few years later that the sex-specific contribution of TLR4 in both inflammatory and neuropathic pain was addressed [183]. It was found that intrathecal administration of LPS induced a robust pain phenotype in male but not female mice, and antagonism of spinal TLR4 reversed this effect in only males. Moreover, neuronal injury induced neuropathic pain behaviors in both male and female mice, but only male mice showed transient attenuation of pain behaviors following intrathecal TLR4 antagonist injection. Some data suggest that the role of TLR4 in inflammatory and neuropathic pain development is reliant on the timing of TLR4 modulation [209, 222]. While another study demonstrated that intrathecal LPS facilitated mechanical hypersensitivity development in both male and female mice, seemingly contrasting findings of TLR4 inhibition were noticed [183]. Inhibition of spinal TLR4 signaling by intracellular antagonist, TAK-242, prevented development of allodynia, but could not reverse established allodynia in males [223]. In accordance with the previously discussed study, another group demonstrated that TLR4 whole-body null females do not exhibit a reduction in mechanical hypersensitivity like their male counterparts after neuropathic injury [224]. This was one of the first studies to highlight that TLR4 either directly or indirectly influences expression of neuronal injury markers in the periphery. While these results seem contradictory, they may be explained by mouse strain differences used [225–228] and serotype and dose of LPS [229–231].

To address discrepancies in the temporal and cell-specific role of TLR4 in chronic pain, a recent study used microglia-specific TLR4 conditional knockout mice, in which a tamoxifen-dependent *Cx3CR1-Cre*^*ERT2−EYFP*^ mouse line was used [232]. They found that systemic antagonism of TLR4 is effective at improving chronic pain outcomes in a tibial fracture model of complex regional pain syndrome in both sexes when administered at the time of injury as opposed to when pain is already established. Their data showed removing microglial TLR4 after the injury resulted in partial improvement in allodynia of both sexes, suggesting that microglial TLR4 plays less of a role in the maintenance of chronic pain states [232]. Interestingly, their investigation showed a female-specific reliance on peripheral TLR4 expressing myeloid cells to trigger chronic pain. In a different context of pain, recent evidence uses a novel null-reactivatable approach that microglial TLR4 is crucial in mediating female-specific acute mechanical allodynia following ethanol exposure [233]. While microglia-restricted TLR4 expression is sufficient to induce allodynia in females, its role in males is less pronounced, underscoring the potential contributions of TLR4 on tissue macrophages in shaping pain responses in both sexes [233]. Differences in microglial pro-inflammatory signatures and nociceptive signaling demonstrate nuanced neuroimmune interactions of central and peripheral TLR4 signaling. This suggests that TLR4 signaling cascades may be different between sexes and temporal dynamics may influence cell-specific actions of TLR4 in the transition from acute to chronic pain.

Other important considerations in studying the effects of TLR4 in chronic pain are the models and inflammatory mediators used. HMGB1 is a highly dynamic nuclear chromatin-binding protein that orchestrates key biological events such as sterile inflammation and chemoattraction based on redox biology [234]. HMGB1 can signal through multiple receptors including TLR4 [235, 236]. Passive secretion of HMGB1 results from cell death, injury, autophagy, and necrosis [237–239]. Intracellular HMGB1 exists in its all-thiol form (at-HMGB1) and can be oxidized to the disulfide form (ds-HMGB1) when reactive oxygen species present in the extracellular matrix facilitate disulfide bridge formation between cysteine residues 23 and 45 [240]. Ds-HMGB1 functions as an inflammatory cytokine that induces preferential activation of TLR4 [241]. HMGB1 is upregulated in sensory neurons in the DRG, as well as peripheral immune cells and glia in response to neuropathic injury [151]. Upregulation of HMGB1 was also associated with a transition from nuclear to cytosolic localization, which is indicative of active release [242]. Inhibition of HMGB1 signaling by a neutralizing antibody prevents mechanical allodynia development and cytosolic localization of the protein, suggesting that peripheral HMGB1 signaling plays a significant role in the development of neuropathic pain [243]. Moreover, only male rats were used in these experiments. Another study demonstrated that administration of ds-HMGB1 increased excitability of dissociated DRG neurons and treatment with glycyrrhizin, an HMGB1 inhibitor, reversed the HMGB1-dependent increase in neuronal excitability. Moreover, systemic inhibition of HMGB1 signaling reversed mechanical allodynia resulting from nerve injury [244]. A more recent study highlighted the effects of central HMGB1-TLR4 signaling in arthritis-like pain [245]. Intrathecal administration of ds-HMGB1 induced pain-like behaviors in mice of both sexes and is TLR4-dependent. Moreover, pharmacological inhibition of spinal HMGB1 signaling reverses arthritis pain. This study provides additional evidence to support the role of HMGB1 in pain, but it was also one of the first to directly assess sex differences in HMGB1-TLR4 signaling [245]. Although, these findings do not recapitulate the Sorge et al., 2011 study that found robust sex differences in spinal TLR4 signaling suggesting mechanistic differences in the specific ligands, signaling pathways, and cell types responsible for neuropathic pain development between males and females [183]. The involvement of sex- and cell-specific HMGB1 signaling in the periphery has not been extensively studied until recently.

A recent study found that inhibition of TLR4 signaling in peripheral macrophages attenuated ds-HMGB1-induced pain behaviors in male but not female mice, suggesting that the cell and tissue types responsible for mediating peripheral pain mechanisms differ between sexes as well [246]. In the same study, using conditional DRG nociceptor TLR4 knockout animals demonstrated that HMGB1 mediates arthritis pain through TLR4 signaling in both sexes. Another study confirmed these findings by using a similar genetic and pain model [247]. Moreover, by conditional deletion of HMGB1 from the whole DRG neuron population, they confirm that HMGB1 is also responsible for neuropathic pain development during neuronal injury in females. The specific receptors for HMGB1, neuronal subtypes, and male-specific mechanisms were not addressed [247]. Therefore, studies that evaluate the effectiveness of inhibiting both TLR4 and HMGB1 signaling will provide insight on the sex- and cell-specific mechanisms of chronic pain and provides a prime example of a mediator of sex-specific differences in neuroimmune interactions.

## Hormonally driven sex differences in neuroimmune communications, pain, and physiology

It is now widely appreciated that the nervous and immune systems communicate with one another to coordinate their dynamic responses. It is also apparent that the endocrine system and hormones also play a role in these actions [248]. The interaction between the nervous, endocrine, and immune systems is bidirectional, indicating that crosstalk between these systems is necessary to not only maintain homeostasis, but also to respond appropriately to tissue injury and infection [36, 248]. While the scientific community has traditionally focused on male-biased research, it is making significant strides to assess differences in nervous and immune system communication between males and females. However, there are still unanswered questions and mechanisms that remain unclear. For instance, TRPV1 activity is well-known for promoting pain and inflammation and can be differentially regulated by different sex steroids. Recent studies found that estradiol levels influence TRPV1 expression in females [249–251], positively modulating its expression to promote pain. However, studies are still inconclusive on whether testosterone modulates TPRV1 expression or activity in humans [252–254]. Studies are still quite sparse regarding sex differences in pain, and thus more studies are needed to unravel the influence of hormones on sex-specific biomarkers driving these dimorphisms.

Accounting for differences between males and females is important as some conditions have a greater prevalence in females compared to males. Moreover, studies confirm that neuroendocrine interactions with the immune system play a role in the pathogenesis of these conditions [255]. For example, males have lower susceptibility to autoimmune disorders compared to females due to testosterone’s protective immunomodulatory function [255, 256]. Studies show that a disproportionate number of females are impacted by autoimmune diseases, chronic pain disorders, and the lack of effective therapeutics [255, 257, 258]. Thus, understanding the sex- and cell-specific differences in neuroimmune modulation is necessary to appropriately design and execute research targeted at effective therapeutic development.

### Testosterone

Androgens have been found to mediate sex differences in neuroimmune interactions and within the last decade, numerous studies have highlighted testosterone as a mediator of sex differences in immunity and pain development [259–261]. Studies have shown that testosterone receptors, also called androgen receptors (AR), modulate immune and pain outcomes in gonadectomized mice by promoting µ-opioid and cannabinoid receptor activity in the presence of high levels of testosterone [262–264]. Another study using a model of chronic inflammatory pain in rats demonstrated that testosterone inhibits TRPV1 expression [265]. Although testosterone serves as the primary sex hormone in males, dysregulation in testosterone production has clinical implications in both sexes. In females, hyperandrogenism is associated with polycystic ovary syndrome (PCOS) [266]. PCOS is an endocrine disorder affecting females of reproductive age and has several associated comorbidities including insulin resistance and cardiovascular disease [267]. Further, in females with PCOS, increased levels of androgens correlate with increased cytokine secretion and disrupts the normal balance of the immune system by stimulating some immune cells and inhibiting others, which can produce a state of chronic, low-grade inflammation [268]. It is especially important that normal-range testosterone levels be maintained in both sexes considering the potential risks associated with immune cardiovascular diseases. A previous study found that administration of testosterone in males with androgen deficiency led to decreased levels of pro-inflammatory cytokines IL-1β and TNF-α and an increase in the anti-inflammatory cytokine IL-10 [269]. Testosterone has also been shown to act directly on CD4^+^ T-cells to increase IL-10 production [256]. Furthermore, research has indicated that testosterone replacement therapy may favorably shift cytokine balance and reduce total cholesterol in males, potentially improving cardiovascular disease risk [269]. Testosterone has also been shown to protect against the development of widespread muscle pain, a hallmark of the female-dominated condition fibromyalgia, in an activity-induced pain model [259]. Hence, more studies unraveling in-depth mechanisms of testosterone in neuroimmune communication are crucial to addressing pain disparities between males and females.

### Estrogens and progesterone

Estrogens are produced mainly by the ovaries in females and to a lesser extent, the testes in males [270]. Estrogens are involved in a variety of physiological processes including the resolution of inflammation and may be a promising therapeutic for promoting anti-inflammation [271]. Fluctuations in estrogens and progesterone during the estrus cycle of female rodents alter the circadian rhythm of both physiology and behavior [272]. The two subtypes of estrogen receptors (ER), ER-α and ER-β, bind estrogens and differentially affect inflammation and pain modulation [273]. ER-α has been shown across multiple studies to modulate pain [275–277]. Estradiol (E2), the most prominent estrogen in the human body, signaling increases NMDA receptor activity while ER-α antagonists administered in the spinal cord increase excitatory postsynaptic currents, which may increase pronociceptive signaling [277, 278]. Further, both male and female ER-α knockout mice show small increases in nociceptive behaviors, suggesting that ER-α signaling inhibits pain transmission [276]. There is evidence, however, that ER-α and ER-β signaling may be pronociceptive, as another study found that knockout of either receptor significantly increases elevated responses to mechanical stimuli in female mice [275]. In most neurons with both receptor subtypes co-expressed, high estrogen levels can result in the dimerization of ER-α and ER-β, which suppresses ER-α signaling [280–282]. In a study looking at how estrogens affect µ-opioid receptor levels and activation with a pain stressor in females, low estrogen levels corresponded with increased pain perception and a reduction in the endogenous opioid neurotransmission function [282]. E2 has been found to mitigate the negative effects of glucocorticoids in cognitive processes [283]. Furthermore, E2 positively activates TRPV1 channels at higher concentrations [249, 251, 284, 285]. The interactions between neurotransmitters like serotonin and estrogens have been linked to female-specific differences in immune modulation and pain perception in conditions like irritable bowel syndrome and fibromyalgia [286]. Serotonin is considered an inflammatory mediator and increases pronociceptive signaling. ER activity can increase serotonin’s pronociceptive activity by enhancing postsynaptic responsiveness and increasing serotonin synthesis in the brain [287].

Although both estrogens and progesterone play a role in modulating pain and inflammation, progesterone’s specific mechanisms are not as well-known as that of estrogen. Progesterone is secreted by the corpus luteum where it has downstream implications in reproductive and immune processes, especially during pregnancy [288]. While progesterone has been extensively studied in relation to female reproductive processes, its role in males is not yet fully understood [289]. Immune cells, including macrophages, NK cells, dendritic cells, and T-cells, express progesterone receptors [290]. Progesterone suppresses macrophage and dendritic cell activation, alters T-cell distribution and activity, and causes apoptosis of NK cells expressing progesterone receptors [290, 291]. Via modulation of transcription factors, progesterone decreases inflammation by inhibiting pro-inflammatory cytokine production and increasing the production of anti-inflammatory cytokines [293–295]. A study investigating progesterone’s anti-inflammatory effects in LPS-stimulated microglia found that progesterone inhibits NF-kB activation [295]. Another study reports that peaks in progesterone and testosterone are associated with lower reported pain severity in females with fibromyalgia, suggesting both sex hormones possess a protective role in modulating fibromyalgia pain [21].

Medications containing hormones, such as oral contraceptive pills containing both estrogens and progestins, can also influence pain and contribute to pain states. These contraceptive pills are the most commonly prescribed contraceptive pills in the United States, however, individuals who experience migraines are not recommended to take them due to the increased risk of migraines [296]. Fluctuations in hormone levels during the menstrual cycle is also believed to play a role in the pathogenesis of migraine attacks and signaling of these hormones may explain the greater prevalence of migraine in females [297].

### Prolactin

In addition to estrogens and progesterone, prolactin, a hormone produced by the pituitary gland and lymphocytes, is also implicated in driving neuroimmune interactions in pain [248, 298]. Prolactin secretion is regulated by cytokines, but can also function as a cytokine itself, with the prolactin receptor (PRLRs) being included in the cytokine receptor super family [299, 300]. Prolactin stimulates PRLRs of B-cells, macrophages, and T-cells to induce immune responses [301]. Prolactin plays a critical role in the complex sex-specific mechanisms underlying migraine [302, 303]. Reports show a direct correlation between high prolactin levels and the prevalence of chronic migraine, with increased risk of migraines in patients with hyperprolactinemia [306–309]. Additionally, a preclinical study showed dural administration of prolactin resulted in female-specific migraine-like behavior in both cycling and ovariectomized rodents [302, 303, 308]. Overexpression of prolactin and its receptors are causal factors in the development of autoimmunity and pain. For example, hyperprolactinemia plays a role in the clinical manifestations of some autoimmune diseases including multiple sclerosis and Sjogren’s syndrome [309]. Furthermore, using both local and spinal inflammatory pain models, a previous study found that PRLR contributes to hypersensitivity in a female-specific manner [310]. Additionally, PRLR has been found to contribute to sexual dimorphic differences in pain during inflammatory processes in neuronal and non-neuronal cells [310, 311]. In a study that used a mouse line with ablated PRLR in sensory neurons, they found that PRLR in sensory neurons is critical for transitioning pain from the acute to chronic state in females only [312]. Additionally, PRLR expression is higher in neuronal fibers and dural mast cells in female mice while male mice showed little to no expression, which may contribute to increased prevalence of migraines in females [308].

### Nuclear receptors: peroxisome proliferator-activated receptors (PPARs)

While hormones and nuclear receptor ligands can contribute to sex differences in neuroimmune communications, they can also serve as modulators of other components that lead to sexual dimorphisms. Peroxisome proliferator-activated receptors (PPARs) are a group of nuclear receptor proteins comprised of three different subtypes (PPARα, PPARγ, and PPARβ/δ). PPARs are involved in glucose metabolism, cell survival, lipid detection, and immunity [313]. While PPARs are expressed in a variety of tissues, the expression of subtypes α and γ in T-cells has been shown to be sexually dimorphic in its contribution to pain and inflammation [314]. PPARα is found in macrophages and lymphocytes where it plays a role in suppressing inflammatory activation [315]. Elevated levels of testosterone in males increase the expression of PPARα, which inhibits IFN-γ, thus suppressing inflammation [316]. A study also found that administration of a PPARα agonist alleviated pain specifically in male mice and this was reversed by eliminating testosterone [166]. Conversely, PPARγ inhibits macrophages and T-cell proliferation and plays a key role in regulatory T-cell activity. Elevated levels of estrogen in females increase PPARγ expression, which inhibits NF-κB and T-cells and suppresses inflammation. Administration of a PPARγ agonist alleviated pain specifically in female mice, and this was reversed by testosterone [166]. Taken together, PPARs and hormones highlight additional mechanisms in which elements of the immune system drive sex differences in pain and inflammation.

## Sex differences in pain and peripheral inflammation in the clinic

The neuroimmune mechanisms underlying sex differences and their impact on chronic pain conditions remains a critical research question for clinical translation. Although animal models provide insight into mechanisms underlying pain, neuroimmune communication, and pain resolution, lack of our understanding of nuances between humans and non-human models slows progress. For example, preclinical rodent models suggest females are less responsive to pain therapeutics, such as low efficacy opioids, however, clinical studies indicate the opposite effect in humans, with opioids generally being more effective and potent in females than males [317, 318]. The severity of the ongoing opioid epidemic is evident in the reported 4 and 5.8-fold increase in overdoses since 1999 in males and females respectively [319, 320]. These increases in overdoses make evident the importance of not only advancing pain research, but also better evaluating the differences across sexes.

Additionally, although existing preclinical and clinical research provides some insight into the sex differences in pain in patients, further investigation is needed to fully elucidate the differences in physiology between males and females to develop and prescribe more personalized, effective pain treatments for patients. Given the higher prevalence of chronic pain conditions in females than in males [321], pain research has honed in on efforts to characterize potential sex differences in actual pain sensitivity and mechanisms [12, 322].

Sex differences in endogenous descending pain modulation have been observed in the form of diffuse noxious inhibitory controls (DNIC). DNIC dampens pain signaling in the dorsal horn of the spinal cord via inhibitory neurotransmitters. Research has found that DNIC is more pronounced in males compared to females [323]. However, as with most clinical data, this evidence is inconsistent due to additional psychological factors rendering DNIC sex differences insignificant [324]. In the clinic, females typically have less favorable outcomes regarding therapeutic intervention than males for pain treatment. In the case of musculoskeletal pain, females are more likely to develop chronic knee pain following total knee arthroplasty and twice at risk of experiencing no clinically important improvement in the case of knee osteoarthritis and widespread pain [325].

The sex differences in pain observed clinically are likely driven by many different cell types and their interactions. For example, immune cells can induce sexually dimorphic pain states through inflammation. In patients with periodontitis, females are more likely to have lower pain thresholds and more persistent pain with macrophage activity likely driving these differences [326]. Furthermore, this study found that genes associated with either promotion or inhibition of nociception differed between sexes [326]. Although the exact immune cells that drive many pain states are still under investigation, sex differences in the levels of pro-inflammatory mediators that drive pain and are involved in the recruitment of immune cells have been reported. For example, in inflammatory dental pain, women show a greater expression in the purinergic receptor, P2X _3_, and CD39, a marker for chronic ATP release, than men, which was indicative of greater pain in women than men [327].Specific immune cells have also been implicated in osteoarthritis in females and males, with osteoarthritis females having a higher percentage of CD4^+^ T cells and macrophages, but a lower percentage of monocytes and CD8^+^ T cells in their synovial fluid compared to osteoarthritis males [328]. These findings indicate that differences in inflammation-related physiology such as pain can be attributed to sex differences.

### Macrophage polarization in asthma and headache related pain

Male and female macrophages differ in propensity to polarize towards pro- and anti-inflammatory states [329, 330]. There is a sex-dependent functional state of immune cells in asthmatic patients, with macrophages and monocytes exhibiting a greater anti-inflammatory M2 phenotype in females [331]. Asthma is also a comorbidity of cluster headaches that is more prevalent in females [332]. There may be a bidirectional etiology where cluster headaches cause further alterations to monocytes, and asthma’s underlying alterations of the immune state may be a contributing factor to cluster headache development [332]. For example, asthma-associated inflammation triggers vascular neuroinflammation to induce headaches, and asthma treatment options are also capable of inducing headaches [333]. While females and males both experience retro-orbital pain with cluster headaches, females disproportionately suffer from coexisting jaw, ear, and cheek pain, as well as migraine-like symptoms such as nausea [332]. Furthermore, sex differences exist in treatment for headache pain, as females respond more to lidocaine and CGRP antagonist, whereas males respond more to sumatriptan [332, 336–338].

### Implications of adiposity and pain

Adiposity serves as a key consideration in the evaluation of risk for obesity, and its sexually dimorphic character contributes to sex disparities in clinical settings, as males and females have uniquely different adipose distributions [337]. Adiposity influences immune responses and has both a direct physical and physiological role in the development of chronic pain. Female adipose distribution is characterized by higher femoral deposition, along with increased levels of inflammatory C-reactive protein (CRP). Moreover, females exhibit higher levels of IL-6 and leptin, a hormone released by adipose tissue, which is implicated in exacerbating chronic pain severity in female patients [338]. Differential plasma levels of inflammatory markers, such as CRP, in females are specifically due to greater accumulation of subcutaneous fat compared to male counterparts [339]. In the case of diet-induced obesity, males and females express distinct inflammatory responses, with males having higher levels of macrophage accumulation in subcutaneous white adipose tissue. This macrophage accumulation is linked to elevated fatty acid species in these tissues, in which their storage and release causes this male-specific inflammation [340]. Preclinical models have demonstrated higher levels of adiposity directly correlate to increased TLR4 expression to mediate pro-inflammatory responses in tissue highlighting another crucial role for TLR4 in the attribution of pain phenotypes in a sex-dependent manner [343–345]. This translates to the clinic where researchers discovered obese patients have high adipocyte TLR4 expression from subcutaneous abdominal fat [344], suggesting TLR4 may play a role in adiposity-related regulation of immune responses and chronic pain.

### Postsurgical recovery and pain

Overall, sex differences in the development of postoperative chronic pain have been identified across many surgery types, with females more frequently reporting severe pain levels one day postoperatively and higher pain scores across the recovery period [345]. Recent studies focused on cardiac surgical outcomes have reported sex differences in postsurgical pain and recovery. In a one year postoperative study, biological females presented with higher levels of postoperative pain than males across time [346], while males had lower mortality rates than females [347, 348]. This indicates that differences in surgical strategy may influence sex differences in recovery outcomes. In total knee arthroplasty procedures, one study found that females experienced more acute postoperative pain, while no sex differences were observed six-weeks postoperatively [349]. Interestingly, this alludes to how biologically, males and females utilize recovery strategies to end up back to homeostasis.

### Sex hormone modulation of nociception

As previously stated, sex hormones and their influence are a culprit behind sexually dimorphic nociceptive responses. However, recent preclinical evidence suggests that transient shifts in hormones like estrogen during the estrus cycle in rodents may not be directly responsible for these nociceptive responses, suggesting estrogen level fluctuations during the menstrual cycle may not be directly responsible for nociceptive responses in humans [35, 176, 350]. It is possible that this dichotomy in sex-based pain may be hardwired at or right before birth and modulated to a lesser degree by the fluctuation of hormones during adulthood. As evidence, a recent clinical study in pre-and full-term infants discovered that widespread nociceptive events measured by electroencephalography are different between sexes [351]. Interestingly, no differences were found in response to innocuous touch, indicating that the signaling pathways between the sensory and nociceptive systems are not mutually exclusive.

### Sex differences in inflammatory factors in osteoarthritis

Many studies have shown that pain in osteoarthritis influences changes in gait [352], and gait changes contribute to pain in a bidirectional manner. Gait differences exist between sexes both pre- and post-surgically in patients with osteoarthritis, indicating that sex differences exist both in inflammatory osteoarthritis development as well as in recovery from associated surgical intervention [353]. Research in osteoarthritis development has gained traction in recent years, taking into consideration that this condition is more prevalent and painful in females [356–358]. Osteoarthritis has been labeled as a condition associated with less inflammation than other arthritis subtypes [356], however, more recent clinical studies have reported sex-differences in the immune response [356–358]. These sex differences have been observed in both the development and in persistent pain in osteoarthritis, however, the exact mechanisms have yet to be fully elucidated [356–358]. Interestingly, females with osteoarthritis show higher levels of IFN-γ, IL-3, IL-8, IL-18, and TNF-β, and other factors that stimulate macrophages compared to osteoarthritis men [357, 358]. Conversely, others report that IL-8 has been found to be higher in males with osteoarthritis [359]. In addition to sex differences in the levels of inflammatory factors in osteoarthritis, these factors may drive pain differently in females and males. For example, IL-6 levels are correlated with increased pain in females and decreased pain in males, IL-1β and IL-8 levels correlate with increased pain in males and decreased pain in females, which further demonstrates the need for sex specific treatments for osteoarthritis [360]. Collectively, these instances of sexual dimorphisms within the clinic emphasize the importance of understanding the influence of sex on neuroimmune interactions and their relation to pain and medicine.

## Conclusions and future perspectives

The neuroimmune “conversation” is implicated in pain physiology, whether through signaling between immune cells and sensory neurons or immune components expressed by neurons. A prime example of bidirectional neuroimmune communication in pain physiology is neuron-microglia crosstalk, in which neurons activate microglia through glutamate or ATP release and activated microglial actions modulate neuronal excitability [142, 144, 145]. Depending on the pain state and the model used, the cell types that drive sexual dimorphisms in pain differ. Sex differences in pain are further influenced by condition and the activity of different cell types, such as microglia in the CNS driving chronic pain development in a sex-specific manner, and by the neuronal expression of prototypic and immune receptors [35]. For example, neuroimmune interactions facilitated by sensory neurons via TLR4 and NLRP3 lead to greater pain sensitivity in females compared to males [151, 152]. The endocrine system and hormones further drive sex differences in neuroimmune communication and pain. Estrogen and progesterone serve as modulators of nuclear hormone receptors, like PPARs, that lead to sexual dimorphisms in immunity and pain. While there is a disproportionate number of females impacted by autoimmune diseases and chronic pain disorders seen clinically, the mechanisms underlying this disparity are understudied. Clinicians and researchers are recognizing the impact of understanding sexual dimorphisms in neuroimmune communication to treat patients more effectively. With this realization, it is paramount that bench-to-bedside approaches are taken with the consideration of the complexities of the individual systems that influence each other, along with the complexities of the human condition. To date, multiple neuroimmune experimental models and clinical findings highlight the influence of sex on physiology and behavior. Females make up ~ 50% of the population, however, until recently, have been excluded from scientific studies and clinical trials that directly impact their health and well-being. It is imperative that studies at all levels include a reasonable representation of sex groups to power statistical analysis to find meaningful results for both sexes. While achieving such numbers, superimposed on the fact that many studies necessitate including several female groups at various stages of the estrus cycle and hormone level determination, makes studies on sex differences expensive and time consuming. However, exclusion of these factors leaves large gaps in our scientific understanding in how pain is driven differently in females and males. Determining the minimum numbers needed for the appropriate statistics will drive research standards and best practices. Elucidating the role of sex differences in neuroimmune communication and pain is essential for the development of efficacious treatments for inflammatory and painful conditions in both sexes.

## Data Availability

Not Applicable.

## Glossary

At-HMGB1: All-thiol form HMGB1
CGRP: Calcitonin gene-related peptide
CIPN: Chemotherapy-induced peripheral neuropathy
CSF1: Colony-stimulating factor 1
DAMPs: Damage-associated molecular patterns
DNIC: Diffuse noxious inhibitory controls
DRG: Dorsal root ganglion
Ds-HMGB1: Disulfide HMGB1
E2: Estradiol
ER: Estrogen receptor
Fc: Receptors for immunoglobulins
HMGB1: High mobility group box 1
LPS: Lipopolysaccharide
M1/2 polarization: Pro-inflammatory (M1) and anti-inflammatory (M2) states
Na_v_: Voltage-gated sodium channel
NK: Natural killer cell
PAMPs: Pathogen-associated molecular patterns
PCOS: Polycystic ovary syndrome
PPARs: Peroxisome proliferator-activated receptor
PRLR: Prolactin receptor
SABV: Sex as a biological variable
TG: Trigeminal ganglion
TLR: Toll-like receptor
TNF-α: Tumor necrosis factor alpha
TRPA1: Transient receptor potential ankyrin 1
TRPV1: Transient receptor potential vanilloid 1
